# *Undaria pinnatifida* Fucoidan Enhances Gut Microbiome, Butyrate Production, and Exerts Anti-Inflammatory Effects in an In Vitro Short-Term SHIME^®^ Coupled to a Caco-2/THP-1 Co-Culture Model

**DOI:** 10.3390/md23060242

**Published:** 2025-06-04

**Authors:** Barbara C. Wimmer, Corinna Dwan, Jelle De Medts, Cindy Duysburgh, Chloë Rotsaert, Massimo Marzorati

**Affiliations:** 1 Marinova Pty Ltd., 249 Kennedy Drive, Cambridge, TAS 7170, Australia; corinna.dwan@marinova.com.au; 2 ProDigest BV, Technologiepark 82, 9052 Ghent, Belgium; jelle.demedts@prodigest.eu (J.D.M.); massimo.marzorati@ugent.be (M.M.); 3 Center of Microbial Ecology and Technology (CMET), Ghent University, Coupure Links 653, 9052 Ghent, Belgium

**Keywords:** fucoidans, *Undaria pinnatifida*, butyrate, gastrointestinal microbiome, SHIME, gut health, immune modulation

## Abstract

Fucoidans have demonstrated a wide range of bioactivities including immune modulation and benefits in gut health. To gain a deeper understanding on the effects of fucoidan from *Undaria pinnatifida* (UPF) on the colonic microbiome, the short-term Simulator of the Human Intestinal Microbial Ecosystem^®^, a validated in vitro gut model, was applied. Following a three-week intervention period on adult faecal samples from three healthy donors, microbial community activity of the colonic microbiota was assessed by quantifying short-chain fatty acids while composition was analysed utilising 16S-targeted Illumina sequencing. Metagenomic data were used to describe changes in community structure. To assess the secretion of cytokines, co-culture experiments using Caco-2 and THP1-Blue™ cells were performed. UPF supplementation over a three-week period had a profound butyrogenic effect while also enriching colonic microbial diversity, consistently stimulating saccharolytic genera, and reducing genera linked with potentially negative health effects in both regions of the colon. Mild immune modulatory effects of UPF were also observed. Colonic fermentation of UPF showed anti-inflammatory properties by inducing the secretion of the anti-inflammatory cytokines IL-6 and IL-10 in two out of three donors in the proximal and distal colon. In conclusion, UPF supplementation may provide significant gut health benefits.

## 1. Introduction

Over the past decade, there has been significant growth in gut health research due to greater understanding of the pivotal role it plays in maintaining human health [1]. Gut dysbiosis, an imbalance or a disruption in the microbial community, has been directly linked to numerous chronic pathologies including inflammatory bowel disease (IBD), obesity, type 2 diabetes, and cardiovascular disease [2]. Furthermore, approximately 20% of all cancer types are thought to be linked to gut microbiota disturbances globally [3].

Dietary sources, such as plant-derived polysaccharides, are gaining attention due to their prebiotic potential and ability to modulate the composition and metabolic function of the microbial community [4,5]. Most polysaccharides cannot be digested and absorbed in the upper gastrointestinal tract and travel to the large intestine where they are fermented by colonic bacteria [6]. Short-chain fatty acids (SCFAs), including acetate, propionate, and butyrate, are the main metabolites of prebiotic fermentation and digestion. These metabolites have many different physiological functions, which influence intestinal health and overall well-being, including regulating luminal pH, fuelling intestinal epithelial cells, improving inflammatory response, stimulating cancer cell apoptosis, and inhibiting pathogenic bacterial growth [2,7].

Natural plant-derived polysaccharides, including those from the marine environment, are particularly attractive given their well-established safety profile, wide-ranging bioactivities, and their potential therapeutic effects [4]. One of the most extensively researched marine polysaccharide groups are fucoidans, highly sulphated polysaccharides found in the cell wall of brown seaweeds [8]. Fucoidans have demonstrated a wide range of bioactivities including antitumour, antioxidant, anti-inflammatory, immunomodulatory, antiviral, antimicrobial, and antithrombic effects [9,10,11]. Additionally, fucoidans have shown potential to improve host health in intestinal disease and bowel health [12]. For example, in an animal model of active ulcerative colitis, oral administration of fucoidan has been shown to significantly reduce inflammatory pathology and clinical symptoms of colitis [13]. More recently, fucoidans have been shown to improve gut microbiome, protect the gastrointestinal tract, and alleviate metabolic syndrome, although the exact mechanisms remain unknown [8,10].

Similar to other prebiotics, clinical effects of fucoidans are not dependent on enzymatic degradation [14] as only small quantities are absorbed in the upper gastrointestinal tract following oral administration [15,16]. Mounting evidence suggests they exert most of their effects on the gut microbiota when reaching the distal colon [8]. Several studies in mice fed a high fat diet demonstrated that fucoidan extracted from *Undaria pinnatifida* (UPF) can positively influence the gut microbiome. In one study, UPF increased the abundance of beneficial gut bacteria, including *Bacteroides/Prevotella*, *Akkermansia muciniphila*, and *Lactobacillus,* while also notably reducing the Firmicutes/Bacteroidetes ratio [17]. In another study, UPF increased the abundance of *Bacteroidetes* and *Alloprevotella*, while also decreasing the abundance of *Staphylococcus* and *Streptococcus* [18]. A further study found that UPF prevented high fat diet-induced obesity, reduced inflammation, and increased gut microbiota abundance [19]. A positive change in gut microbiota along with an increase in SCFAs was observed in fibre-deficient mice who received UPF [20]. This modulation decreased fibre deficiency-induced inflammation by reducing the levels of inflammation-related factors, decreasing oxidative stress and lipid abnormality [20]. Consistent with previous research, rats fed a high fat diet and supplemented with UPF were found to have significant improvements in gut microbiota, serum dyslipidaemia, bile salt hydrolase, and bile acid metabolism-related pathways [21]. Most of these studies were conducted in special populations such as animals with high fat diet or fibre deficiency.

Although informative, most available microbiome data on the effects of UPF have come from animal studies, despite rodent microbiome being significantly different from humans [8]. The present study used an adapted Simulator of the Human Intestinal Microbial Ecosystem (SHIME^®^) model to investigate the effects of repeated daily doses of UPF on colonic microbiome, derived from human faecal samples. This is a standardised system which has been used extensively in the past [22,23,24]. Additionally, the immune modulation effects of UPF were assessed using co-cultures of Caco-2-cells and THP-1 macrophages [25]. This study found that ongoing UPF supplementation can positively alter the gut microbial composition, promote butyrate production, and exert immune modulatory and anti-inflammatory effects through anti-inflammatory cytokines. These effects may contribute to health-promoting outcomes.

## 2. Results

### 2.1. UPF Effect on SCFAs

**Figure 1:**
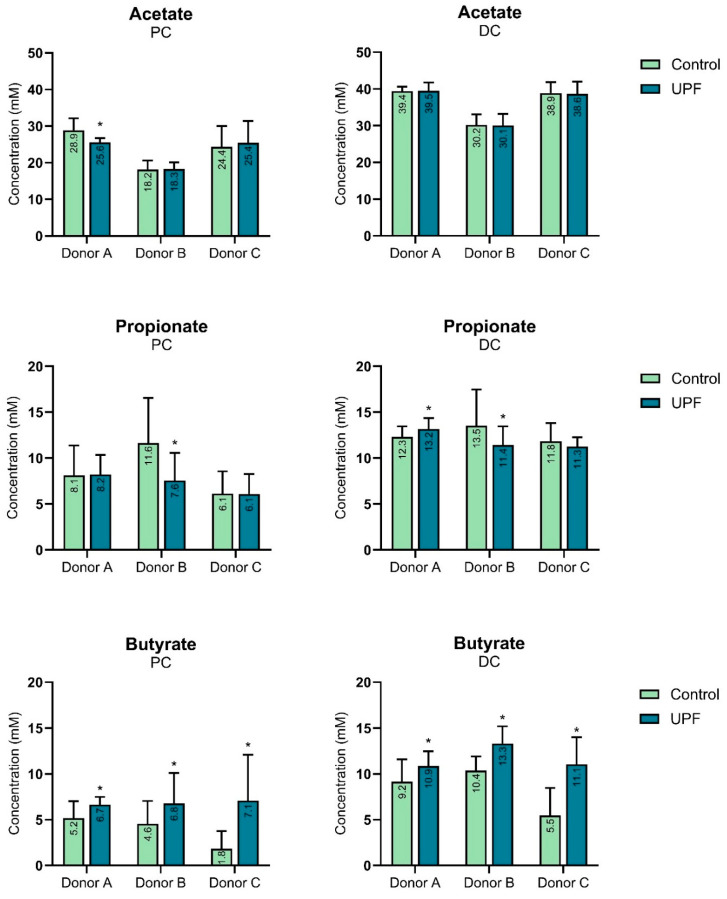
Effect of UPF versus control on acetate, propionate, and butyrate levels in the proximal and distal colon for donors A, B, and C. * indicates statistically significant difference between treatment and control for each donor at *p* < 0.05. DC: distal colon, PC: proximal colon.

Overall, acetate levels remained largely unaffected upon UPF supplementation in the proximal and distal colon of all donors, except for a reduction in concentration for donor A in the proximal colon (Figure 1). Inter-donor variation in propionate levels was observed upon supplementation of UPF for all donors, with levels significantly reduced for donor B in both colon regions, while levels were enhanced in the distal colon of donor A (Figure 1). In contrast, butyrate levels were significantly increased in both the proximal and distal colon upon UPF supplementation for all donors (Figure 1). Mean concentration increase ± STDEV for each SCFA are provided in the Supplementary Information: Supplementary Table S1.

### 2.2. UPF Effect on Microbial Diversity

**Figure 2:**
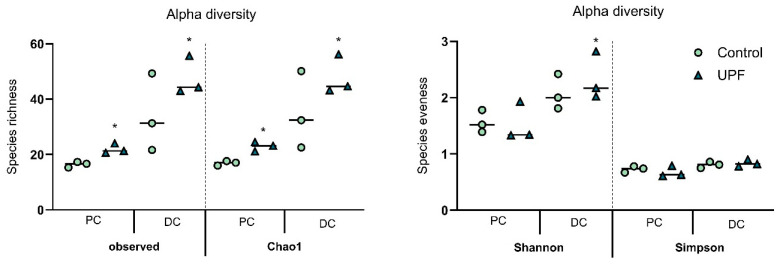
Effect of UPF on alpha-diversity, as calculated in observed taxa, Chao1, and the Shannon and Simpson indices in the proximal and distal colon during the last week of treatment period compared to the control, for three donors tested. Indices are based on relative abundances. For each index, at week 3 of treatment, the weekly average was calculated across the three timepoints for each donor (*n* = 3), along with the average across the three donors (*n* = 9 with *n* = 3 per donor). * indicates statistically significant differences between control and UPF (*p* < 0.05). DC: distal colon, PC: proximal colon.

The effect of UPF on alpha-diversity in the proximal and distal colon was assessed for the three donors, as well as across donors. Four different alpha-diversity measures were calculated, consisting of observed taxa, Chao1 index, Shannon index, and Simpson index (Figure 2). Following UPF supplementation, observed taxa and Chao1 indices across donors showed significant increases in both colon regions, resulting in enhanced species richness. Furthermore, a significant stimulation of the Shannon index was observed across donors in the distal colon. Moreover, the Shannon and Simpon indices were significantly higher upon treatment in the distal colon of donor B. Both indices translate to an effect of the treatment on species evenness, which indicates that UPF positively impacted bacterial distribution.

The numerical average relative abundance values are provided in Table 1.

**Figure 3:**
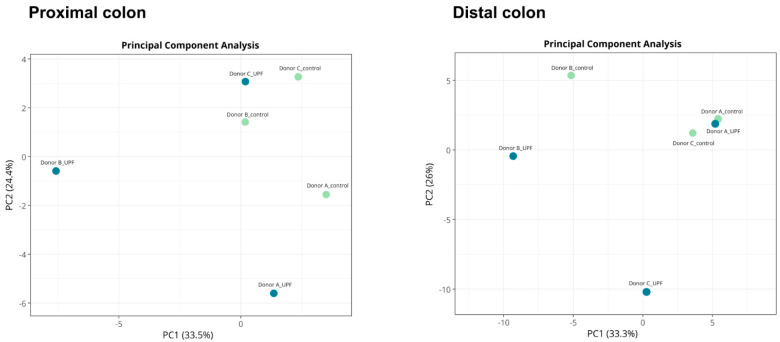
Differences in community composition (beta-diversity) in the proximal and distal colon during the last week of the treatment period, following treatment with UPF compared to a control for three donors tested. Analysis is based on relative abundances. Unit variance scaling is applied to rows; Singular Value Decomposition with imputation is used to calculate principal components. X- and Y-axes show principal component 1 and principal component 2 that explain % of the total variance. Each dot represents the weekly average calculated across three timepoints for each donor (*n* = 3).

Differences in beta-diversity assessed by Principal Component Analysis (PCA) showed a clear effect of UPF supplementation on microbial diversity (Figure 3). In the proximal colon, shifts along the principal components were observed for all donors upon treatment, with strongest effects observed for donor B as shown by the biggest shift along the first principal component. In the distal colon, shifts were observed along the principal components for donor B and C, while treatment and control clustered together for donor A suggesting communities were more similar in this donor.

### 2.3. UPF Effect on Microbial Community Composition

**Figure 4:**
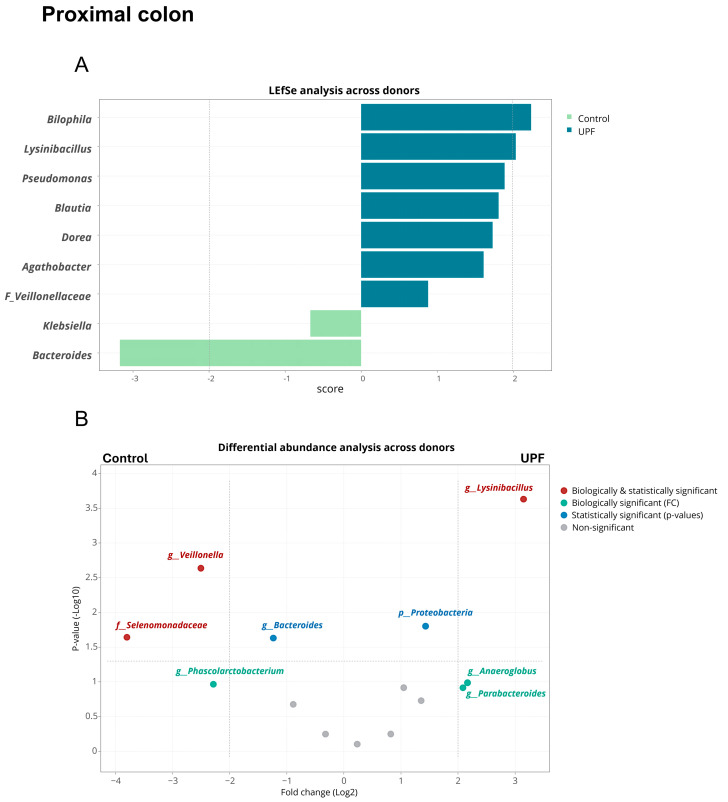
Linear discriminant analysis of effect size (LEfSe), shown as histogram and differential abundance analysis (treeclimbR), for the effects of treatment with UPF (**A**) versus control (**B**) across donors (*n* = 3) in the proximal colon.

Linear discriminant analyses of effect size (LEfSe) and treeclimbR analysis were used to identify the bacterial groups responsible for changes observed in community composition in the colonic environment. LEfSe and treeclimbR identified genera that were significantly different between UPF supplementation and the control across donors and within donors in the proximal and distal colon. Across donors, supplementation with UPF was associated with statistically and biologically significant enrichments of *Bilophila* and *Lysinibacillus* in the proximal colon according to LEfSe and of *Lysinibacillus* according to treeclimbR (Figure 4). However, within donors, inter-donor variation was observed, with no enhancement or reduction in *Bilophila* in donors A and C found, while the effect in donor B was large enough to evoke a significant result across donors (Refer to Supplementary data Figure S1 for individual donor results).

*Pseudomonas*, *Blautia*, *Dorea*, *Agathobacter*, an unidentified member within the *Veillonellaceae* family, and the *Proteobacteria* phylum were statistically significantly enriched by UPF without biological significance being reached, while *Anaeroglobus* and *Parabacteroides* were enriched on a biologically relevant level without reaching statistical significance. *Bacteroides*, *Veillonella*, and the *Selenomonadaceae* family were biologically and statistically reduced upon UPF treatment, while *Klebsiella* was statistically and *Phascolarctobacterium* biologically reduced.

**Figure 5:**
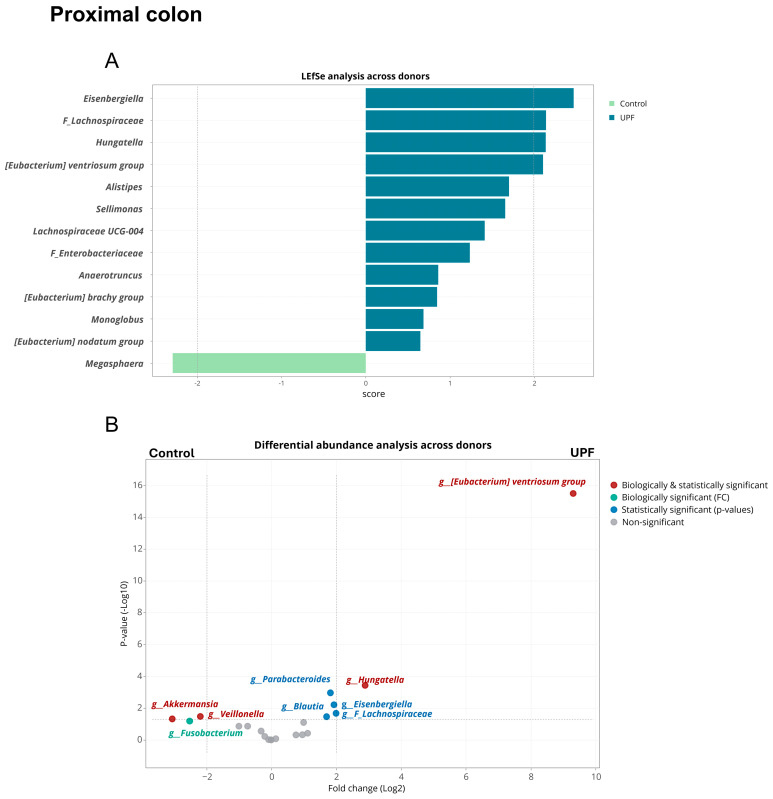
Linear discriminant analysis of effect size (LEfSe), shown as histogram and differential abundance analysis (treeclimbR), for the effects of treatment with UPF (**A**) versus control (**B**) across donors (*n* = 3) in the distal colon.

In the distal colon, across donors, the *Eisenbergiella*, *Hungatella*, and *Eubacterium ventriosum* group, as well as an unidentified genus within the *Lachnospiraceae* family, were biologically and statistically enhanced by UPF supplementation, according to LEfSe and/or treeclimbR analysis (Figure 5). Additionally, *Alistipes*, *Sellimonas*, *Lachnospiraceae UCG-004*, *Anaerotruncus*, *Monoglobus* and an unspecified genus within the *Enterobacteriaceae* family were statistically but not biologically enhanced by UPF supplementation.

*Megasphaera*, *Akkermansia*, and *Veillonella* were statistically and biologically significantly reduced by treatment with UPF, while *Fusobacterium* was reduced on a biologically but not statistically significant level. However, within donors, *Akkermansia muciniphila* abundance was highly donor-dependent; UPF supplementation resulted in reduced *Akkermansia* abundance for donor C, while remaining unaffected for donor A (Refer to Supplementary Figure S2).

### 2.4. UPF Effect on Immune Markers

**Figure 6:**
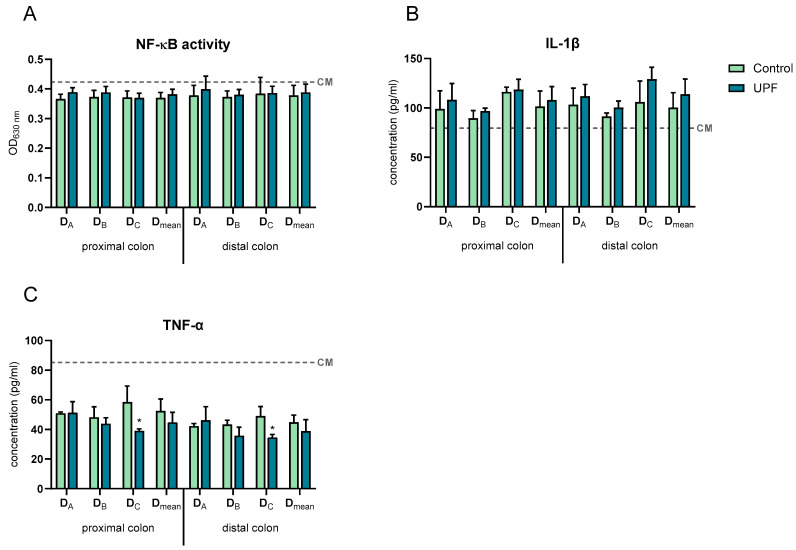
Effects of treatment with UPF versus control on basolateral NF-κB activity of THP1-Blue™ cells (**A**), basolateral secretion of IL-1β (**B**), and TNF-α (**C**). Levels were measured 24 h after treatment of the Caco-2/THP1-Blue co-cultures. The dotted line corresponds to the CM. Data are plotted as mean ± STDEV. D_A_–D_C_ = donor A to donor C; D_mean_ = average of all 3 donors. * indicates statistically significant differences between the control and treatment samples (*p < 0.05*).

After 24 h incubation, all SHIME samples showed a slight decrease in nuclear factor kappa-light-chain-enhancer of activated B cells (NF-κB) activity and an increase in pro-inflammatory cytokine interleukin (IL)-1β compared to the complete medium control (CM) (Figure 6). However, in both colon regions of all donors, UPF supplementation did not significantly affect NF-κB activity or IL-1β compared to their respective controls.

A decrease in the secretion of the pro-inflammatory cytokine tumour necrosis factor alpha (TNF-α) compared to the CM control was observed in all SHIME samples after 24 h (Figure 6). Additionally, in both the proximal and distal colon, UPF supplementation significantly decreased TNF-α secretion in donor C compared to the control. Across donors, no significant differences were observed between UPF and control in both colon compartments.

**Figure 7:**
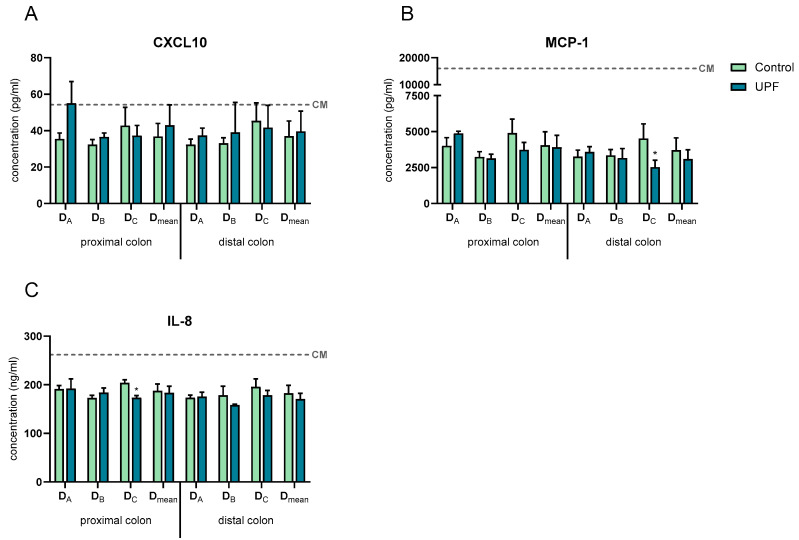
Effect of SHIME suspensions on basolateral secretion of CXCL10 (**A**), MCP-1 (**B**), and IL-8 (**C**). Levels were measured 24 h after treatment of the Caco-2/THP1-Blue co-cultures. The dotted line corresponds to the experimental control CM (complete medium). Data are plotted as mean ± STDEV. * indicates statistically significant differences between the control and treatment samples (*p* < 0.05).

All SHIME samples decreased the secretion of the pro-inflammatory chemokines C-X-C motif chemokine ligand 10 (CXCL10), monocyte chemoattractant protein-1 (MCP-1), and IL-8 compared to the CM, except for the proximal colon treatment samples of donor A on CXCL10. Furthermore, treatment with UPF did not significantly affect CXCL10 secretion compared to the control in both the proximal and distal colon of all donors. In contrast, in donor C, treatment with UPF significantly reduced IL-8 secretion in the proximal colon and MCP-1 secretion in the distal colon compared to their controls. However, across donors, no significant differences in CXCL10, MCP-1, and IL-8 secretion were observed upon treatment compared to the control (Figure 7).

**Figure 8:**
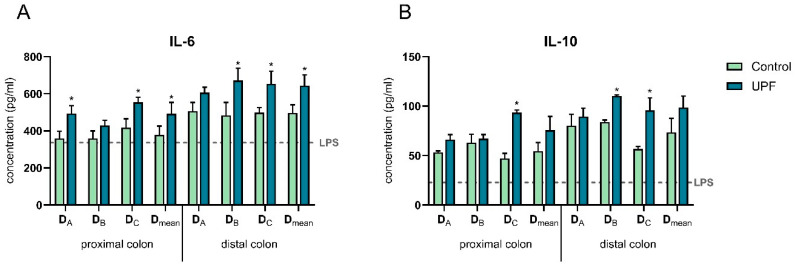
Effect of SHIME suspensions on basolateral secretion of IL-6 (**A**) and IL-10 (**B**). Levels were measured 6 h after LPS treatment on the basolateral side of the Caco-2/THP1-Blue co-cultures after pre-treatment of the apical side for 24 h with SHIME suspensions. The dotted line corresponds to the experimental control LPS+. Data are plotted as mean ± STDEV. * indicates statistically significant differences between the control and treatment samples (*p* < 0.05).

All SHIME samples increased the secretion of the anti-inflammatory cytokines IL-6 and IL-10 compared to the lipopolysaccharide (LPS)+ control (Figure 8). Furthermore, in the proximal colon, treatment with UPF induced IL-6 and IL-10 secretion in donors A and C, reaching significance compared to the control in donor C for both IL-6 and IL-10 secretion and in donor A for IL-6 secretion. In the distal colon, treatment significantly increased the secretion of the anti-inflammatory cytokines IL-6 and IL-10 in donors B and C compared to their control. Across donors, a significant increase in IL-6 levels was observed upon treatment with UPF in both the proximal and distal colon.

## 3. Discussion

This exploratory study aimed to investigate the effect of repeated UPF dosing on the colonic microbiome of three healthy adult donors compared to controls, using the validated in vitro short-term SHIME^®^ model. Results showed that UPF was able to be fermented by the microbial community of the three adult hosts. Importantly, a strong butyrogenic effect following repeated daily administration of UPF over a three-week period was observed for all donors in both colon regions. This significant enhancement in butyrate levels may be related to the stimulation of *Agathobacter* and *Anaeroglobus* in the proximal colon and *Eubacterium ventriosum* and *Eisenbergiella* in the distal colon, as revealed in the 16S sequencing data. *Agathobacter*, a member of the *Lachnospiraceae* family [26], and *Anaeroglobus* from the *Veillonellaceae* family [27] are commensal bacteria with the ability to produce lactate, acetate, and importantly, butyrate. Similarly, species in the *Eisenbergiella* genus are also known to produce butyrate, lactate, and acetate [28]. In contrast, acetate levels remained largely unaffected, while inter-donor variation in propionate levels was observed upon UPF supplementation in the proximal and distal colon regions.

The strong butyrogenic effect upon UPF supplementation is significant given that butyrate plays such an important role in gut health. It is the primary energy source for colonocytes; it also helps to maintain gut barrier, stimulate gut motility, and modulate both innate and adaptive immune responses [29,30]. Furthermore, the production of butyrate through the gut microbiome has been shown to have a strong influence on the peripheral immune system. These immune cells communicate with the central nervous system and can affect brain activity [31]. Additionally, butyrate has been shown to regulate inflammatory responses by inhibiting NF-κβ activation in the colonocytes, decreasing macrophage secretion of pro-inflammatory cytokines, and increasing anti-inflammatory cytokines [29,30,32].

Lower butyrate concentrations and a reduced number of butyrate-producing bacteria are associated with chronic conditions such as IBD [30]. This has led to a growing interest in exploring the therapeutic potential of butyrate in individuals with ulcerative colitis (UC) and Crohn’s disease. However, recent clinical trial results with encapsulated butyrate and enemas used as adjuvant therapy were mixed and limited in scope. Further studies are required to elucidate its potential; however, the unpleasant taste and odour of butyrate may make oral administration challenging; hence, dietary ingredients that can enhance butyrate levels may be useful in this context [32]. Interestingly, fucoidan in a previous study was shown to positively influence UC-related inflammatory pathology, but the underlying mechanisms were unknown [13]. This study suggests positive changes in butyrate levels may have contributed to its effect.

Repeated treatment with UPF also had a significant impact on microbial diversity; species richness and species evenness were positively enhanced in both the distal and proximal colon. Supplements that can positively affect microbial diversity may be particularly useful in supporting individuals with ill health, as this population tends to have reduced microbial diversity, which in turn can impede immune function response [33,34]. Interestingly, a correlation with decreasing faecal microbial diversity has previously been identified for biological but not chronological age [33]. In physically frail older people, a decrease in microbiome diversity was detected, while interventions aimed at increasing microbial diversity showed positive effects in decreasing inflammatory markers and frailty [35]. Similarly, patients with IBD also tend to have lower microbial diversity than healthy individuals [34,36]. Therefore, nutritional ingredients that can enhance microbial diversity may be beneficial in such populations.

Consistent with other dietary fibres, fucoidans’ main effects are exhibited in the lower digestive tract, especially the distal colon area [8]. In this study, Illumina 16S sequencing provided an in-depth analysis of the microbial community. In the distal colon, treatment with UPF resulted in statistically and biologically significant elevated levels in the *Eisenbergiella*, *Hungatella*, and *Eubacterium ventriosum* group and an unidentified genus from the *Lachnospiraceae* family. The significant increase in *Eubacterium ventriosum* is interesting as this taxon is typically reduced in patients with colorectal cancer (CRC) and, like *Eisenbergiella,* is also known to be a butyrate producer [37,38]. A low prevalence of *Eubacterium ventriosum* has been suggested as a potential biomarker for CRC [38]. Additionally, a patent on the therapeutic use of *Eubacterium ventriosum* in CRC indicates a possible clinical benefit [37]. Furthermore, an increasing number of experimental studies suggest butyrate may play a positive role in the prevention and inhibition of CRC [32]. In vitro models have shown butyrate can inhibit the proliferation of tumour cells, reduce carcinogen-induced DNA damage, and induce apoptosis [29,32].

Both *Eubacterium ventriosum* and *Hungatella* have been found to be associated with depressive symptoms, but in different directions [39]; higher levels of *Hungatella* were found in patients with depressive symptoms [40], while decrease in *Eubacterium ventriosum* has been shown to occur in both patients with traumatic brain injury and in those with depressive symptoms [39]. In this study, the greatest increase was observed in abundant *Eubacterium ventriosum*. One explanation for the relationship between these taxa and depressive symptoms was proposed to be their effect on glutamate, serotonin, gamma-aminobutyric acid (GABA), and butyrate production [39]. The association between SCFA levels and mood disorder symptoms is gaining more traction and interest [41]. Research suggests an involvement of SCFAs in mood disorders and depressive symptoms, with more research on mechanistic involvement needed.

In this study, differential abundance analyses found a biologically and statistically significant reduction in *Veillonella* in the proximal and distal colon. While the prevalence of *Veillonella* in the intestine is beneficial to some parts of the human body due to its capabilities to produce acetate and propionate out of lactate, some *Veillonella* species are associated with infectious diseases, and the genus has a positive correlation with IBD [42]. In non-obese patients with non-alcoholic fatty liver disease, *Veillonellaceae* were strongly associated with liver fibrosis, leading authors to suggest this family as a diagnostic microbial biomarker [43]. A systematic review of the literature found evidence of higher levels of the genus *Veillonella* in patients with Crohn’s disease [44]. Additionally, since IBD patients seem to have a higher prevalence of *Veillonella* spp. and *Prevotella* spp. than healthy controls [45], a decrease in these two genera may be beneficial in patients with this condition. However, more research is needed to confirm this correlation.

In the proximal colon, a statistically and biologically significant enhancement of *Bilophila* and *Lysinibacillus* was observed. The clinical relevance of this increase is not clear. *Lysinibacillus* is generally found in soil and aquatic environments and not much is known about its effects on the gastrointestinal tract. *Bilophila* spp., and more specifically *Bilophila wadsworthia*, is a sulphate-reducing bacterium that metabolises taurine in the production of hydrogen sulphide. While hydrogen sulphide plays a regulatory role in gut homeostasis, excessive levels can contribute to inflammation and damage the intestinal lining [46]. In this study, however, the increase in *Bilophila* was attributed to the increase in one participant (donor B), while no enhancements were observed in the other two donors.

Additionally, it has been suggested that fucoidan can exert inhibitory effects on Ca^2+^ responses via different pathways and that the polysaccharide may inhibit different G-protein-coupled receptors associated with Ca^2+^ dynamics. Fucoidans’ effects on ion channels are considered to involve both direct interaction with the channels and indirect modulation of cellular processes that can influence ion channel activity [47]. This is interesting because it is known that ion transport influences the luminal environment, and therefore, one mechanism of UPF modulating the gut microbiota composition could be through potential effects on ion channels [48].

Immune modulatory effects of fucoidans including the induction of IL-6 have been widely reported [9]. These data stem from in vitro, in vivo, and clinical studies. Consistently, immune modulatory properties were also observed in this study upon colonic fermentation of UPF. Treatment induced the secretion of anti-inflammatory cytokines IL-6 and IL-10 in two out of three donors in the proximal and distal colon and decreased the secretion of pro-inflammatory cytokine TNF-α in both colon regions of one out of three donors. More specifically, anti-inflammatory effects were observed by significantly inducing the secretion of the anti-inflammatory cytokine IL-10 in both the proximal and distal colon of donor C and in the distal colon of donor B. In addition, the secretion of IL-6 (both anti- and pro-inflammatory properties) was significantly increased in the proximal colon of donors A and C and in the distal colon of donors B and C. While IL-6 overexpression can contribute to chronic inflammation, it is also a key player in gut homeostasis, and it can support the resolution of acute inflammation by promoting neutrophil apoptosis. IL-6 is crucial for epithelial cell proliferation and the preservation of the stem cell niche, both essential for intestinal wound healing [49]. Depending on the timing and context of upregulation, IL-6 plays a complex role in the gut [50]. In addition, in donor C, colonic fermentation of UPF demonstrated anti-inflammatory properties in terms of reducing the secretion of the pro-inflammatory cytokine TNF-α in the proximal and distal colon and in reducing the secretion of the chemokines IL-8 and MCP-1 in the proximal and distal colon, respectively.

The main limitation of this study is that the observed results cannot be directly translated to an in vivo biological response. This is the case for any in vitro study; however, the short-term SHIME is a well-established model running over an extended period simulating physiological conditions. In a previous in vitro study utilising intestinal microbial culture medium containing human faeces samples from healthy donors, fucoidan significantly increased SCFA concentrations [51]. However, the brown seaweed species from which this fucoidan extract was sourced was not mentioned in the paper, and samples were only collected up to 48 h of fermentation. Another study investigated the effect of gut fermentation on a polysaccharide from *Fucus vesiculosus* including associated microbial changes and changes in SCFAs. This in vitro study also utilised faecal fermentation for up to 48 h, with the authors reporting an increase in SCFAs and associated microbial species [52]. Both above-mentioned fermentation periods are significantly shorter than in the current study, where investigations were conducted for over three weeks, simulating long-term repeated administration of the test compound, as would occur in a physiological environment. Another potential limitation of the study includes the limited assessment of inter-individual variations, including only three healthy human donors. However, it is important to note that the human gut microbiome is generally characterised by large differences in terms of community composition [53], and this should therefore be taken into account upon using in vitro gut models. Further studies are thus needed to confirm fucoidan’s prebiotic properties in clinical studies on gut health. Also, including donors with different health conditions could be explored in follow-up studies, given that the observed effects in the current research could theoretically be interesting under certain disease conditions.

In conclusion, donor-dependent effects were observed, but overall, UPF supplementation resulted in a pronounced butyrogenic effect, positive changes in bacterial distribution, and the combination of colonic microbial composition with mild-anti-inflammatory effects. This process occurred along the entire lower digestive tract, which is of particular interest as many colonic diseases occur in the distal colon areas. While some donor-dependent stimulations could be observed on specific bacterial groups of interest (e.g., *Bifidobacterium*, Bacteroidetes, and Firmicutes), 16S-targeted sequencing showed consistent stimulation of saccharolytic genera (e.g., *Parabacteroides* and *Eisenbergiella*), while reducing genera linked with potential negative health effects (e.g., *Veillonella*). Overall, the prebiotic effects of UPF may have health-promoting effects in modulating host microbiome. These effects need to be verified and investigated further in human studies focusing on gut health and immune modulation, while also considering inter-individual variations.

## 4. Materials and Methods

### 4.1. Ethics

Faecal samples for this specific study were collected according to the ethical approval received from the Ethics Committee of the University Hospital Ghent (reference number ONZ-2022-0267) and stored in a biobank (registration number BB220010).

### 4.2. Faecal Sample Donor Description

Human healthy donors (*n* = 3; donor A: F, 34 years; donor B: F, 28 years; donor C: F, 56 years) were selected based on the following inclusion criteria: healthy, aged between 18 and 65 years, no antibiotic intake during the last 4 months prior to donation, typical Western diet, no history of chronic disease, no constipation, and no pre- or probiotic intake. Faecal inoculum was prepared as a 1:5 mixture of freshly collected faecal sample and anaerobic phosphate buffer. After homogenisation and removal of big particles via centrifugation, 5% inoculum was added to the different colon compartments.

Informed consent of the donors was obtained after providing them with detailed information on the study and sample use.

### 4.3. Materials Used

All chemicals were obtained from Sigma-Aldrich (Overijse, Belgium) unless stated otherwise. UPF (Batch No. UPF2022555) with >85% concentration (dry weight) was extracted according to current good manufacturing practices (cGMP) by Marinova Pty Ltd. (Cambridge, Tasmania, Australia). The proprietary aqueous extraction and filtration process has been specifically designed to extract fucoidan without the use of organic solvents. Fucoidan purity was calculated as the sum of total carbohydrates, sulphation, acetylation and cations. For the carbohydrate profile analysis, a gas chromatography method was used to assess individual monosaccharide ratios. Total carbohydrate content was determined spectrophotometrically using the phenol–sulphuric technique [54], while uronic acid content was determined by spectrophotometric analysis in the presence of 3-phenylphenol [55]. Sulphate content was determined spectrophotometrically using a BaSO4 precipitation technique [56]. The chemical composition of fucoidan used in this study is described in Table 2.

A UPF dose of 1.5 g was administered daily for three weeks as intervention, which is equivalent to a 3 g daily dose in humans, resulting in a target concentration of 2.5 g/L in the colon model. This dose was used because 3 g of UPF has previously been administered successfully in clinical trials [16,57,58].

The control group was defined as the conditions in which no intervention product was added for each of the donors.

### 4.4. Experimental Design of the Adapted Short-Term SHIME^®^ Model

**Figure 9:**
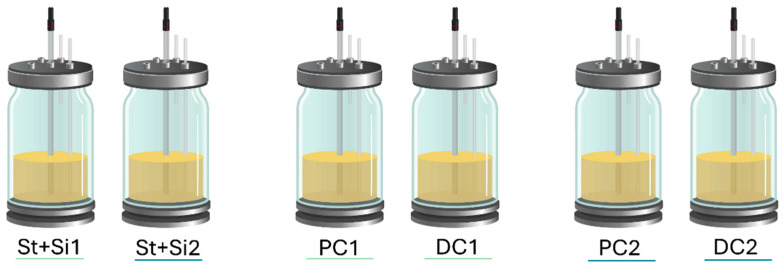
Experimental design. L-SHIME^®^ configurations were used to assess the impact of one treatment condition (1) and one control (2) (2 arms per donor) on the gut microbiome of each individual donor. In total, three donors were tested (resulting in a total of three L-SHIME^®^ configurations). St + Si: vessel serving as stomach and small intestine, PC: proximal colon and DC: distal colon.

The adapted short-term SHIME^®^ in vitro model (ProDigest-Ghent University, Ghent, Belgium), with the applied volumes mimicking half a human, was used to evaluate the effect of UPF repeat dosing on the colonic microbiome of three healthy donors. The reactor configuration for this study was adapted from Molly et al. [59], as validated previously [60,61,62] and recently reviewed by Zhu et al. [63], and contained two consecutive reactors to simulate the proximal and distal regions of the colon. A control arm (without test product addition) was included for each donor for comparison (Figure 9).

Upon inoculation with adult faecal microbiota, retention times and pH ranges were optimised to resemble in vivo conditions in the proximal colon (PC operating at a pH of 5.75–5.95 with a retention time of 20 h) and the distal colon (DC operating at a pH 6.60–6.90 with a retention time of 32 h).

The short-term SHIME^®^ setup consisted of an inoculation period and a treatment period. For the inoculation period, the colon reactors, which were continuously stirred, were inoculated with appropriate faecal samples and allowed to grow and colonise the reactors. After overnight inoculation, the colon reactors were fed with the standard nutritional medium for two additional days to support maximum diversity of the gut microbiota originally present in the faecal inoculum. This also allowed the microbial community to be differentiated in the reactors depending on the local environmental conditions.

After inoculation, a 3-week treatment period followed. During this time, the SHIME^®^ reactor was fed 3×/day with the standard SHIME^®^ nutritional medium [autoclaved SHIME^®^ nutritional medium (15.6 g/L PDNM001B (ProDigest); 70 mL downscaled volume per feeding cycle; set at pH = 2.0) and pancreatic juice (12.5 g NaHCO_3_ (Thermo Fisher Scientific, Merelbeke–Melle, Belgium), 6 g/L ox gall (Thermo Fisher Scientific), and 0.9 g/L pancreatin; 30 mL downscaled volume per feeding cycle)]. In the treatment arm, this diet was supplemented with UPF during each feeding cycle.

### 4.5. Microbial Community Activity Analysis

Microbial community activity of the colonic microbiota was assessed by quantifying SCFAs. Quantitative analysis of SCFA concentrations, including acetate, propionate, and butyrate was conducted by capillary gas chromatography coupled with a flame ionisation detector [64]. The isolation of SCFA was performed by liquid–liquid extraction using acetonitrile as the extract solvent. Specifications of the method included a linear dynamic range of 0.05–50 mM and limit of detection (LOD) of 0.02 mM. Each measurement was performed in single repetition. Fermentation samples were collected three times per week (Monday–Wednesday–Friday) from each colon vessel during the treatment period.

### 4.6. Microbial Community Composition Analysis

At the end of the treatment period, samples were collected from each colonic vessel three times (Monday, Wednesday, Friday) for quantitative 16S-targeted Illumina sequencing. The average of these three samples was recorded as the weekly average. DNA was isolated as described by Boon et al. [65], with modifications as described by Duysburgh et al. [66]. DNA was extracted from the pellet of 1 mL sample (obtained upon centrifugation for 5 min at 9000× *g*). The original genomic DNA extracts were diluted in DNase/RNase/protease-free water (Thermo Fisher Scientific Bvba) to obtain a concentration of 50 ng/µL, and 30 µL was sent for 16S-rRNA gene profiling by LGC Genomics GmbH (Berlin, Germany).

Sequencing and flow cytometry methods followed established protocols [67]. 16S-targeted Illumina sequencing involves primers that span 2 hypervariable regions (V3-V4) of the 16S rRNA gene, i.e., 341F (5′-CCTACGGGNGGCWGCAG-3′) and 785R (5′ GACTACHVGGGTATCTAAKCC-3′), resulting in 424 bp amplicons via paired-end sequencing. Amplicon sequence data were processed with the DADA2 R package [68], including primer removal, trimming (truncQ = 2), quality filtering (maxEE = 2.2), dereplication, and denoising with the DADA algorithm (pooling = TRUE). After error rate inspection, denoised reads were merged. Finally, after chimaera removal, the ASV table obtained was used for taxonomy assignment using the Naive Bayesian Classifier and the DADA2 formatted Silva v138.1 [69]. The proportional phylogenetic information (%) was combined with total cell quantification obtained via flow cytometry. Flow cytometry samples were diluted in Dulbecco’s Phosphate-Buffered Saline (DPBS) and stained with 0.01 mM SYTO24 (Life Technologies Europe, Merelbeke, Belgium) for 15 min at 37 °C. The samples were then analysed on a BD Accuri C6 Plus (BD Biosciences, Erembodegem, Belgium) at high flow rate. Bacterial cells were differentiated from medium debris and signal noise by applying two threshold values: a primary FSC-H threshold of 200 and a secondary FL-1 threshold of 700. Flow cytometry data were analysed using FlowJo software, version 10.9.0.

### 4.7. Immune Modulation Analysis

Samples were collected at the end of each colonic vessel and used in an in vitro Caco-2/THP1 co-culture model, consisting of human intestinal epithelial and monocyte/macrophage cells, to investigate the effects of UPF after fermentation on immune markers (pro- and anti-inflammatory cytokines and chemokines). The Caco-2/THP-1 co-cultures were based on the procedure previously described, including slight adaptations [25,70].

Caco-2 cells (HTB-37; American Type Culture Collection, LGC Promochem, Molsheim, France) at passage 47 were seeded into 24-well semi-permeable inserts (0.4 µm PET translucent CellQart^®^, Sabeu, Northhelm, Germany) at a density of 1 × 10^5^ cells/insert. Cells were cultured for 14 days with three medium changes/week until a functional cell monolayer with a transepithelial electrical resistance (TEER) above 300 Ω·cm^2^ was obtained. TEER was measured using an Epithelial Volt-Ohm meter Millicell ERS-2 (Merck Millipore, Overijse, Belgium). Cells were maintained in Dulbecco’s Modified Eagle Medium (DMEM) containing 25 mM glucose and 4 mM glutamine and supplemented with 10 mM HEPES and 10% (*v*/*v*) heat-inactivated foetal bovine serum (FBS; Life Technologies). Prior to co-culture, THP1-Blue™ cells (InvivoGen, Toulouse, France) at passage 26 were seeded into 24-well plates at a density of 5 × 10^5^ cells/well and treated with phorbol 12-myristate 13-acetate (PMA) for 48 h. THP1-Blue™ cells were maintained in Roswell Park Memorial Institute (RPMI) 1640 medium containing 11 mM glucose and 2 mM glutamine and supplemented with 10 mM HEPES, 1 mM sodium pyruvate, and 10% (*v*/*v*) heat-inactivated FBS.

Caco-2-bearing inserts were then placed atop the PMA-differentiated THP1-BlueTM cells. The apical compartment (containing the Caco-2 cells) was filled with sterile-filtered (0.22 µm) colonic suspensions. Cells were also treated apically with sodium butyrate as a positive control. The basolateral compartment (containing the THP1-Blue™ cells) was filled with Caco-2 complete medium. Cells were also exposed to Caco-2 complete medium in both chambers as the control.

After 24 h, the basolateral supernatants were collected to measure secretion of the pro-inflammatory cytokines (IL-1β, TNF-α) and chemokines (CXCL10, MCP-1 and IL-8) via Luminex^®^ multiplex (Thermo Fisher Scientific, Merelbeke, Belgium) as well as NF-κB activity using the QUANTI-Blue™ reagent (Invivogen). THP1-Blue™ cells, which contain a stably transfected reporter construct, express secreted alkaline phosphatase (SEAP) under NF-κB inducible promoter control. Upon toll-like receptor (TLR) activation, these transcription factors were activated, leading to SEAP expression and secretion, which was then measured in the basolateral supernatant. The THP1-Blue™ cells contain a stably transfected reporter construct allowing the expression of a secreted alkaline phosphatase (SEAP) under the control of an NF-κB inducible promoter. Hence, upon LPS stimulation, NF-κB was activated, leading to the secretion of SEAP in the basolateral medium. Then, SEAP activity was measured in the basolateral medium using the QUANTI-Blue™ reagent (Invivogen). Subsequently, cells were stimulated at the basolateral side with Caco-2 culture medium containing ultrapure LPS (InvivoGen) or medium without LPS (CM; complete medium) as control. After 6 h of LPS stimulation, the basolateral supernatants were collected to measure secretion of the anti-inflammatory cytokines (IL-6 and IL-10) by Luminex^®^ multiplex. All experiments were conducted in triplicate and cells were incubated at 37 °C in a humidified atmosphere of air/CO_2_ (95.5 *v*/*v*).

### 4.8. Data Processing and Statistics

For each SCFA parameter, an unpaired two-tailed homoscedastic *t*-test was used to compare different experimental weeks with each other and this for each of the test conditions separately. In addition, a comparison of UPF intervention with its respective control was performed using a two-tailed paired t-test for each donor. Differences were considered statistically significant if the *p*-value was <0.05.

For 16S-targeted Illumina sequencing data, bacterial differences in alpha-diversity, beta-diversity, and differential abundance analysis were conducted to examine the effects of UPF in each colon region for the three donors, as well as across donors compared to the control.

Alpha-diversity: The observed taxa, Chao1, Shannon, and Simpson diversity indices were calculated using phyloseq v1.44.0 [71]. To evaluate whether differences in alpha-diversity between conditions were statistically significant, paired two-sided *t*-tests were performed in which each timepoint was considered a replicate measurement. An effect was considered significant if the obtained *p*-value was <0.05.

Beta-diversity: PCA was used to investigate whether fucoidan administration affected the overall community composition. The relative abundance of all genera within the gut microbiome was calculated and uploaded onto online software programme Clustvis.

Differential abundance analyses on relative abundance data were performed using two statistical methods, LEfSe [72] and treeclimbR [73]. Both were obtained by total sum scaling.

By measuring the extent and statistical significance of differences in bacterial abundances between two conditions, LEfSe enables identification of treatment-induced community shifts. To do so, the algorithm couples statistical significance with biological consistency and effect size estimation, thus providing an in-depth insight into the biological relevance and magnitude of bacterial enrichments. All features shown in the LEfSe plots meet *p* ≤ 0.05 for Kruskal–Wallis and Wilcoxon tests. LDA scores express the extent of differences in taxon abundances between conditions. The higher the LDA score, the higher the difference in abundance between the two biological conditions. LDA scores ≥ 2.0 are considered biologically relevant.

TreeclimbR analysis was performed to identify the taxa most likely to explain differences between treatment and control, and the outcome was plotted on a volcano plot. The volcano plot shows statistical significance (adjusted *p*-value, y-axis) versus magnitude of change (fold change, x-axis). Bacterial enrichments exceeding a fold change of 4 (log_2_4 = 2 in chart) as compared to control were considered biologically relevant; the cut-off for statistical significance was set at a *p*-value of 0.05 (or −log_10_0.05= 1.3 on the *y*-axis). Therefore, bacterial enrichment with −log(*p*-value) > 1.3 was considered statistically significant.

For immune modulation analysis, to evaluate differences in immune markers at the individual donor level, treated samples were compared to their non-treated controls using a two-way ANOVA with Sidak’s multiple comparisons test. To assess differences in immune markers between treatment and control on the average of donors, paired two-tailed *t*-tests were performed, using the individual donors as replicates. Differences were considered statistically significant if the *p*-value was <0.05.

Statistical analysis for the cell assays was performed using GraphPad Prism version 10.4.1 for Windows (GraphPad Software, San Diego, CA, USA) or via excel (*t*-tests).

## Figures and Tables

**Table 1:** Four alpha-diversity measures were calculated: (1) ‘Observed’ taxa (measure for richness), (2) ‘Chao1’ (measure for richness), (3) ‘Shannon’ (measure for richness and evenness), and (4) ‘Simpson’ (measure for richness and evenness, giving more weight to common or dominant species).

Alpha-Diversity		Observed	Chao1	Shannon	Simpson
Proximal colon	Control	16.44 ± 1.81	16.92 ± 2.05	1.56 ± 0.19	0.73 ± 0.06
	UPF	**22.00 ± 3.46**	**22.94 ± 3.37**	1.53 ± 0.37	0.68 ± 0.12
Distal colon	Control	34.11 ± 13.29	35.06 ± 13.08	2.08 ± 0.29	0.81 ± 0.06
	UPF	**47.67 ± 10.69**	**48.02 ± 10.75**	**2.34 ± 0.40**	0.83 ± 0.06

Statistically significant differences between control and UPF (*p* < 0.05) are depicted in bold.

**Table 2:** Characterisation of *Undaria pinnatifida* fucoidan extract.

Fucoidan Extract		% Total Sugars		% Uronic Acids		% Sulphate		% Fucoidan (Dry Weight)	
UPF2022555		58.34		4.69		29.90		86.9	
**Fucoidan** **Extract**	**As (ppm)**	**Cd (ppm)**	**Fucose**	**Xylose**	**Mannose**	**Galactose**	**Glucose**	**Arabinose**	**Rhamnose**
UPF2022555	7.22	1.62	44.1	0.8	4.3	45.1	2.9	1.6	1.2

## Data Availability

The raw data supporting the conclusions of this article will be made available by the authors on request.

## Supplementary Materials

The following supporting information can be downloaded at: https://www.mdpi.com/article/10.3390/md23060242/s1: Figure S1. LEfSe and treeclimbR of individual donors in the proximal colon. Figure S2. LEfSe and treeclimbR of individual donors in the distal colon. Table S1. Mean concentration increase ± STDEV for each SCFA in the proximal and distal colon for donors.
