# Supplementary material for: Undaria pinnatifida Fucoidan Enhances Gut Microbiome, Butyrate Production, and Exerts Anti-Inflammatory Effects in an In Vitro Short-Term SHIME® Coupled to a Caco-2/THP-1 Co-Culture Model
Source: Mar Drugs. 2025 Jun 4;23(6):242. doi: 10.3390/md23060242 (PMC12194508; doi:10.3390/md23060242)
Supplement: Supplementary file 1 [file marinedrugs-23-00242-s001.zip › Supplementary material.pdf]

**Supplementary material**

**Supplementary Table 1:** Effect of UPF versus negative control on production of short-chain fatty acids acetate, propionate and butyrate in the proximal and distal colon for donors A, B and C. Samples were collected in triplicate and data are reported as mean ± STDEV

| Acetate | | Donor A | Donor B | Donor C |
|---|---|---|---|---|
| Proximal colon | Control | 28.87±3.27 | 18.15±2.45 | 24.36±5.64 |
| | UPF | 25.55±1.15 | 18.25±1.86 | 25.44±5.96 |
| Distal colon | Control | 39.43±1.23 | 30.21±2.89 | 38.9±2.95 |
| | UPF | 39.52±2.27 | 30.07±3.14 | 38.64±3.4 |

| Propionate | | Donor A | Donor B | Donor C |
|---|---|---|---|---|
| Proximal colon | Control | 8.12±3.25 | 11.64±4.91 | 6.14±2.42 |
| | UPF | 8.22±2.14 | 7.55±3.02 | 6±2.18 |
| Distal colon | Control | 12.3±1.15 | 13.54±3.94 | 11.84±1.99 |
| | UPF | 13.17±1.2 | 11.43±2.02 | 11.25±1.02 |

| Butyrate | | Donor A | Donor B | Donor C |
|---|---|---|---|---|
| Proximal colon | Control | 5.18±1.85 | 4.55±2.51 | 1.82±1.94 |
| | UPF | 6.65±0.84 | 6.78±3.32 | 7.09±5.02 |
| Distal colon | Control | 9.16±2.45 | 10.38±1.53 | 5.46±3.02 |
| | UPF | 10.87±1.61 | 13.33±1.87 | 11.06±2.94 |

# Proximal colon

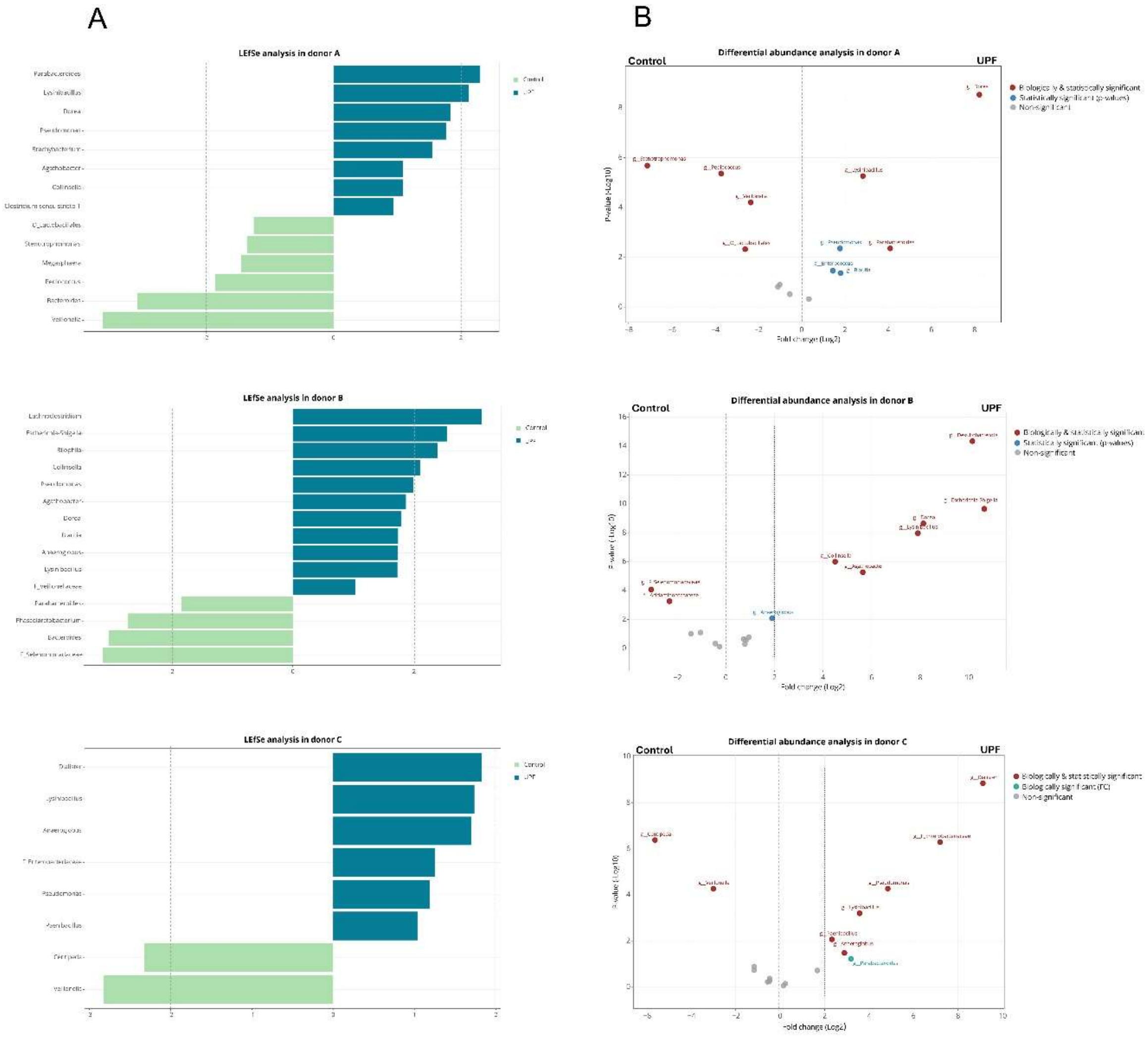


**Supplementary Figure 1**: Linear discriminant analysis of effect size (LEfSe) shown as histogram and Differential abundance analysis (treeclimbr) for the effects of treatment with UPF versus negative control for donor A, donor B and donor C in the proximal colon.

## Distal colon

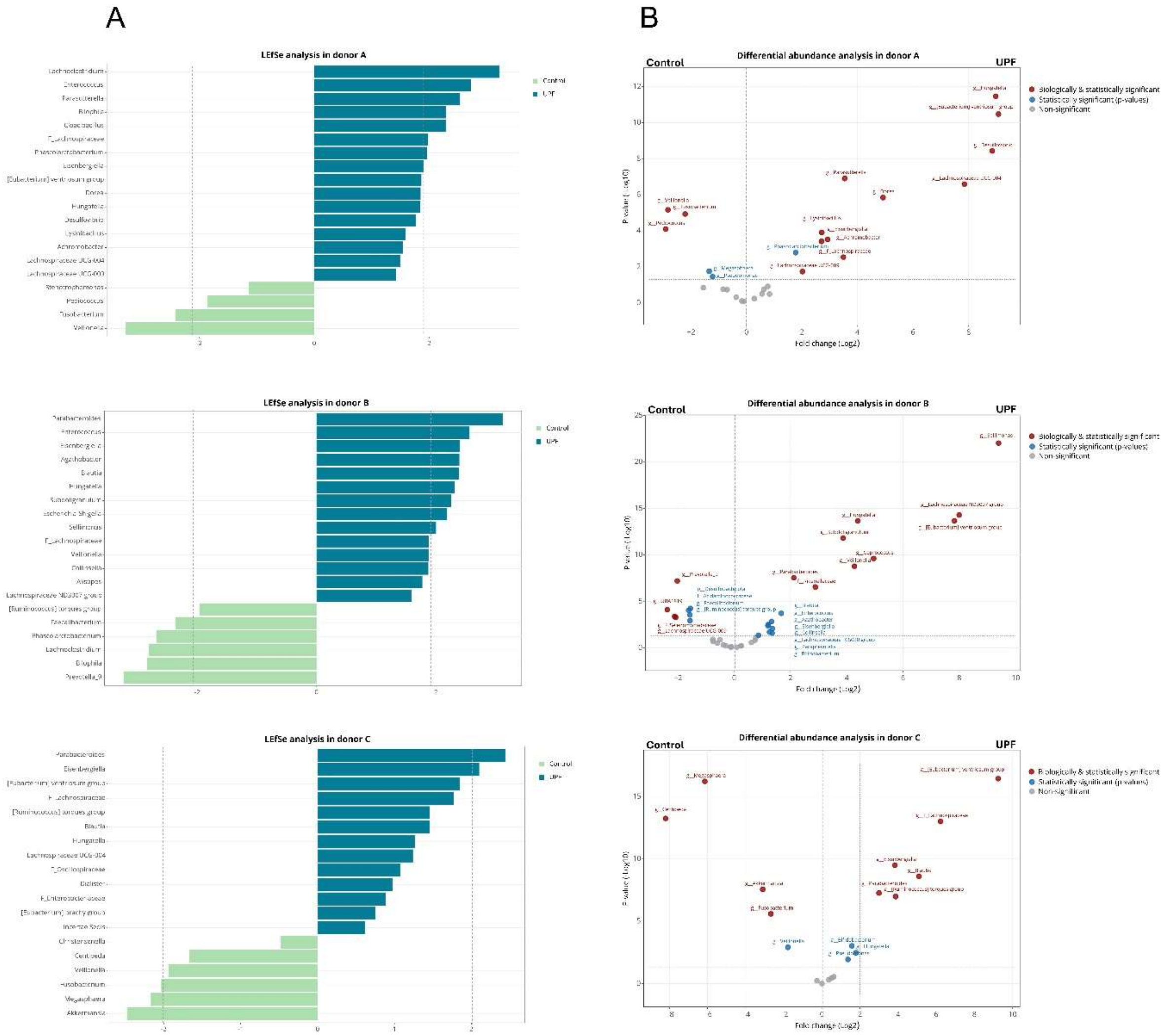


**Supplementary Figure 2**: Linear discriminant analysis of effect size (LEfSe) shown as histogram and Differential abundance analysis (treeclimbr) for the effects of treatment with UPF versus negative control for donor A, donor B and donor C in the distal colon.
